# Wearable-Sensor-Based Classification Models of Faller Status in Older Adults

**DOI:** 10.1371/journal.pone.0153240

**Published:** 2016-04-07

**Authors:** Jennifer Howcroft, Edward D. Lemaire, Jonathan Kofman

**Affiliations:** 1 Department of Systems Design Engineering, University of Waterloo, Waterloo, Canada; 2 Centre for Rehabilitation, Research and Development, Ottawa Hospital Research Institute, Ottawa, Canada; 3 Faculty of Medicine, University of Ottawa, Ottawa, Canada; Tianjin University, CHINA

## Abstract

Wearable sensors have potential for quantitative, gait-based, point-of-care fall risk assessment that can be easily and quickly implemented in clinical-care and older-adult living environments. This investigation generated models for wearable-sensor based fall-risk classification in older adults and identified the optimal sensor type, location, combination, and modelling method; for walking with and without a cognitive load task. A convenience sample of 100 older individuals (75.5 ± 6.7 years; 76 non-fallers, 24 fallers based on 6 month retrospective fall occurrence) walked 7.62 m under single-task and dual-task conditions while wearing pressure-sensing insoles and tri-axial accelerometers at the head, pelvis, and left and right shanks. Participants also completed the Activities-specific Balance Confidence scale, Community Health Activities Model Program for Seniors questionnaire, six minute walk test, and ranked their fear of falling. Fall risk classification models were assessed for all sensor combinations and three model types: multi-layer perceptron neural network, naïve Bayesian, and support vector machine. The best performing model was a multi-layer perceptron neural network with input parameters from pressure-sensing insoles and head, pelvis, and left shank accelerometers (accuracy = 84%, F1 score = 0.600, MCC score = 0.521). Head sensor-based models had the best performance of the single-sensor models for single-task gait assessment. Single-task gait assessment models outperformed models based on dual-task walking or clinical assessment data. Support vector machines and neural networks were the best modelling technique for fall risk classification. Fall risk classification models developed for point-of-care environments should be developed using support vector machines and neural networks, with a multi-sensor single-task gait assessment.

## Introduction

Falls are a serious health concern for the elderly, with 30% of individuals older than 65 years falling each year [1], costing approximately 20 billion dollars a year in the United States [2]. Approximately half of these falls occur during walking activities [3]. After experiencing a fall, fear of falling can reduce activities of daily living, leading to physical deterioration, social isolation, and decreased quality of life [1,4].

Predicting fall risk would allow earlier interventions for fall risk reduction [5]. Wearable sensors have potential for quantitative, gait-based, point-of-care fall risk assessment that can be easily and quickly implemented in clinical-care and older-adult living environments. A wide variety of wearable-sensor, inertial-based variables have been used to predict and classify fall risk with varying levels of success (accuracy: 62–100%, specificity: 35–100%, sensitivity: 55–99%) [6]. A detailed review of fall-risk assessment using inertial sensors is given in Howcroft [6]. Gait data is a cyclic, time-series set (i.e., repeated steps). Network techniques have been used in other healthcare applications with time-series data sets; such as, pathological heartbeat detection [7], ventricular fibrillation detection [8], and detection of pathological brain dynamics [9–11]. Broader, non-health care applications include turbulence [12], flow [13,14], and chaotic [15] dynamics analysis.

While wearable-sensor-based fall risk prediction and classification have had some success, optimized prediction and classification models that consider sensor type (e.g. accelerometer, gyroscope, pressure-sensing insole), sensor placement (e.g. head, pelvis, sternum, ankles, shoes), and model type (e.g. neural network, naïve Bayesian, decision tree, support vector machine, logistic regression) [6] are required. Some studies compared sensor-based fall risk predictive and classification capabilities to clinical questionnaire and assessment-based fall risk predictive and classification capabilities [16–19] and most found that adding wearable sensor data to the model improved fall risk prediction and classification compared to models based only on clinical data [16–18]. However, no study has compared model performance using different sensor types, sensor body locations, or combinations of sensors. Furthermore, few studies [20–22] have assessed different model types to optimize fall risk classification and predictive capabilities.

This paper presents a comprehensive investigation of fall-risk classification capabilities that included two types of wearable sensors (accelerometers, pressure-sensing insoles), four accelerometer locations (head, pelvis, left and right shank), and three types of models (neural network, support vector machine, naive Bayesian). Furthermore, the effect of cognitive demand on fall risk classification was assessed using single-task (ST) and dual-task (DT) gait. The objectives of this study were to: (1) identify the best wearable-sensor type, location, and combination for faller status classification (faller or non-faller), (2) determine whether single-task or dual-task gait is more effective for faller status classification, and (3) determine if models based on wearable-sensor gait measurement outperform models based on clinical assessment for older-adult faller classification.

## Methods

### Participants

A convenience sample of 100 people, 65 years or older, were recruited from the community (Table 1). Participants were identified as fallers if they reported at least one fall during the six months prior to study participation. Potential participants were excluded if they had a cognitive disorder (self-reported) or were unable to walk for six minutes without an assistive device. The University of Waterloo, Office of Research Ethics approved the study and all participants gave informed written consent.

**Table 1:** Participant characteristics.

	Participants (#)	Age (years)	Height (cm)	Weight (kg)	6MWT distance (m)
Fallers	13 male, 11 female	76.3±7.0	165.2±10.3	71.9±14.3	446.6±101.4
Non Fallers	31 male, 45 female	75.2±6.6	165.1±9.9	73.1±13.4	455.8±102.4

### Protocol

Participants reported six month retrospective fall occurrence, age, and sex. Body weight and height were measured. Participants completed the Activities-specific Balance Confidence (ABC) scale [23] and Community Health Activities Model Program for Seniors (CHAMPS) [24] questionnaires. They also ranked their fear of falling from 0 (no fear) to 10 (high level of fear).

Pressure-sensing insoles (F-Scan 3000E, Tekscan, Boston, MA) were equilibrated using multi-point calibration (137.9, 275.8, 413.7 kPa), fit to the shoes, and calibrated. Accelerometers (X16-1C, Gulf Coast Data Concepts, Waveland, MS) were attached to the posterior head with a band, posterior pelvis with a belt, and lateral shank, just above the ankle, with a band. Plantar pressure data were collected at 120 Hz and accelerometer data at 50 Hz.

In separate trials, the time to complete a 7.62 m (25 ft) walk with (dual task: DT) and without (single task: ST) a cognitive load was recorded. The cognitive load was a verbal word fluency task requiring the participants to say words starting with A, F, or S [25]. Participants also completed the six minute walk test (6MWT) under standard, ST conditions [26]. The starting letter and order of walking activities were randomized.

### Data Processing

**Fig 1:**
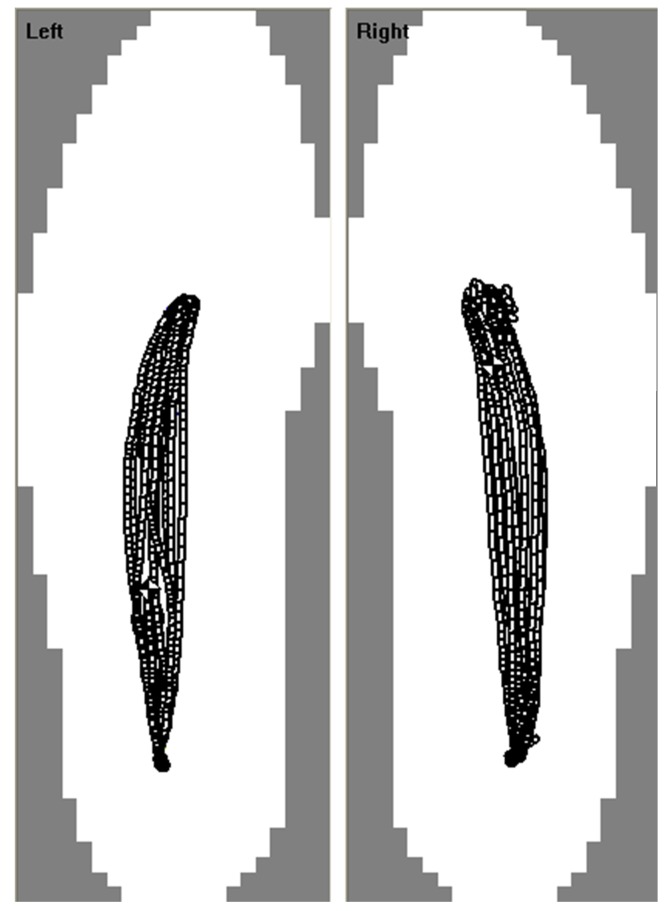
Plantar pressure derived CoP path for 10 ST gait strides.

**Fig 2:**
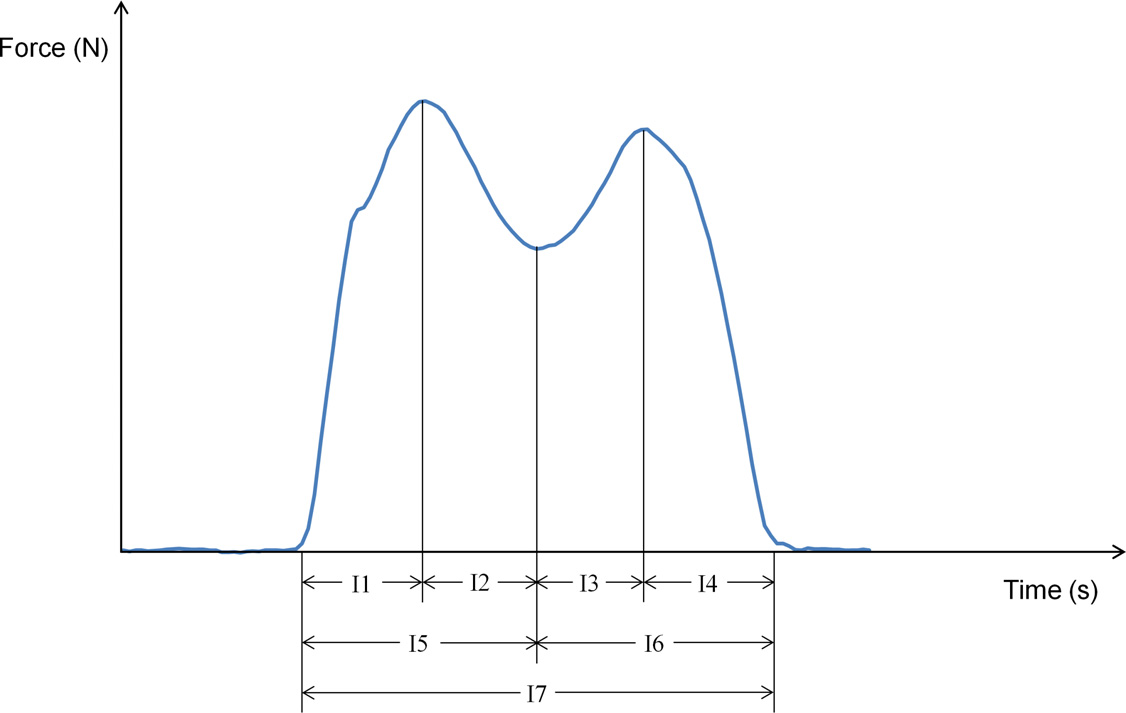
Typical total ground reaction force curve with impulse phases indicated.

Gait velocities for ST and DT trials were calculated as 7.62 m divided by the time. Plantar-pressure and accelerometer data were exported to Matlab v2010a to calculate outcome variables for the 7.62 m ST and DT trials. Thirty plantar-pressure derived parameters were:

- Center of Pressure (CoP) path (Fig 1): Since the CoP path should advance monotonically and anteriorly, posterior CoP path movements were identified as irregular. The number, length, and duration of posterior deviations (PD) per stance phase were determined. Similarly, smooth medial and lateral movements were expected. Deviations were defined as the first derivative of the CoP ML signal exceeding a dual threshold of ± 0.5 mm/frame [27]. The number, length, and duration of ML path deviations per stance were determined. Minimum, maximum, mean, and median CoP path velocities were also calculated and normalized by stance time. AP and ML coefficients of variation (CoV) for the stance phase CoP path were calculated by determining the mean and standard deviation of CoP path positions at 1% intervals, determined using ensemble averaging [28], for the entire stance phase and calculating the overall CoP path stance phase CoV as in Winter [29].
- Temporal: Cadence, stride time, stance time, swing time, percent stance time, percent double support time, stride time symmetry index [30] between the left and right limbs, and CoV for stride time, stance time, and swing time.
- Impulse: Impulse variables were determined from the total force-time curve (sum of forces from all insole sensels, Fig 2) and calculated based on the area under the force-time curve normalized by body mass (Ns/kg) for: I1 (foot-strike to first peak), I2 (first peak to minimum), I3 (minimum to second peak), I4 (second peak to foot-off), I5 (foot-strike to minimum), I6 (minimum to foot-off), and I7 (foot-strike to foot-off).

All variables were calculated for each stride for the left and right limbs for each walking condition (ST and DT) before calculating means and standard deviations across both limbs (i.e. left and right limb combined).

**Fig 3:**
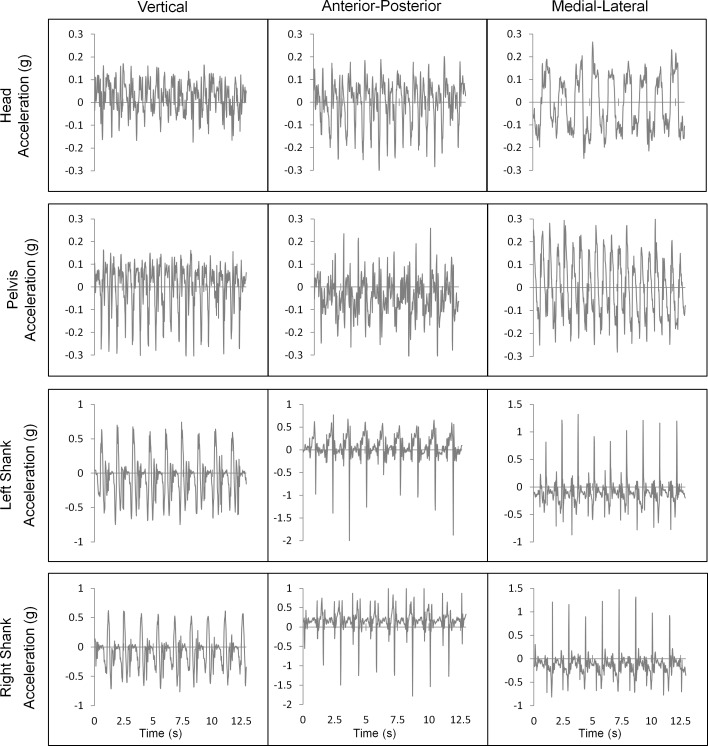
ST gait accelerations. Vertical: positive is upwards, AP: positive is anterior, ML: positive is toward participant’s right.

For accelerometer data (Fig 3), the positive vertical axis was upwards, positive AP axis was anterior, and positive ML axis was toward the participant’s right. Accelerometer-derived parameters were:

- Descriptive statistics: Maximum, mean, and standard deviation of acceleration for the superior, inferior, anterior, posterior, right, and left axes.
- Temporal features: Cadence and stride time.
- Fast Fourier Transform (FFT) Quartile: Percentage of acceleration frequencies in the first quartile (i.e., frequencies ≤ 12.5 Hz) of an FFT frequency plot for vertical, AP, and ML axes.
- Ratio of even to odd harmonics (REOH): Proportion of the acceleration signal in phase with stride frequency. The harmonic ratio is used to measure irregular accelerations and overall gait pattern stability [31–33]. The harmonic ratio was calculated for vertical, AP, and ML axes as in Smidt [34].
- Maximum Lyapunov exponent (MLE): Average rate of expansion or contraction of the original trajectory in response to perturbations [35,36], calculated for vertical, AP, and ML accelerations, as in van Schooten [37]. The number of dimensions was determined using the global false nearest neighbours method [38] and a fixed time delay based on the first minimum of the average mutual information [39].

For descriptive statistics and MLE parameters, acceleration data were filtered using a fifth order, low pass Butterworth filter with a 12.5 Hz cut-off frequency. Unfiltered acceleration data were used to calculate the FFT quartile and REOH.

### Model Development

Three classifier models were assessed for fall-risk classification capability: multi-layer perceptron neural network (NN), naïve Bayesian (NB), and support vector machine (SVM). Retrospective fall occurrence was the classification criterion. For all models, 75% of participant data (18 fallers, 57 non-fallers) were used for training and 25% were used for testing (6 fallers, 19 non-fallers). Pelvis accelerometer data were missing for two non-fallers and left shank accelerometer data were missing for one non-faller due to sensor power failure. All models were developed with the Matlab R2010a standard model algorithms. The Neural Network Pattern Recognition Toolbox was used for NN development and supervised backpropagation training was performed using the Neural Network Training tool. NN with 5, 10, 15, 20, and 25 nodes in a single hidden layer were evaluated. Neural networks between the best NN and the best of the two neighbouring NN were also evaluated. For example, if the 15-node NN provided the best classification and the 20-node NN outperformed the 10-node NN, NN with 16, 17, 18, and 19 nodes were also evaluated. Other models included linear and quadratic multinomial NB models, and SVM with polynomial kernels with degrees one to seven.

Fall classification models were based on all gait variables derived from the wearable sensors, separately for ST and DT gait data. All possible sensor combinations (Table 2) were evaluated using all 138 parameters (30 pressure insole parameters, 29 accelerometer parameters at 4 body locations). In addition, models were developed with clinical assessment data: ABC score, CHAMPS derived activity frequency and calorie expenditure, 6MWT distance, ST and DT walk times, fear of falling levels.

**Table 2:** Summary of sensor combinations and total number of input parameters.

Sensor Combination	Sensor Description	Total parameters
I	pressure insole	30
H	accelerometer (head)	29
P	accelerometer (pelvis)	29
LS	accelerometer (left shank)	29
RS	accelerometer (right shank)	29
H-P	accelerometer (head, pelvis)	58
H-LS	accelerometer (head, left shank)	58
H-RS	accelerometer (head, right shank)	58
P-LS	accelerometer (pelvis, left shank)	58
P-RS	accelerometer (pelvis, right shank)	58
LS-RS	accelerometer (left shank, right shank)	58
H-P-LS	accelerometer (head, pelvis, left shank)	87
H-P-RS	accelerometer (head, pelvis, right shank)	87
H-LS-RS	accelerometer (head, left shank, right shank)	87
P-LS-RS	accelerometer (pelvis, left shank, right shank)	87
H-P-LS-RS	accelerometer (head, pelvis, left shank, right shank)	116
I-H	pressure insole; accelerometer (head)	59
I-P	pressure insole; accelerometer (pelvis)	59
I-LS	pressure insole; accelerometer (left shank)	59
I-RS	pressure insole; accelerometer (right shank)	59
I-H-P	pressure insole; accelerometer (head, pelvis)	88
I-H-LS	pressure insole; accelerometer (head, left shank)	88
I-H-RS	pressure insole; accelerometer (head, right shank)	88
I-P-LS	pressure insole; accelerometer (pelvis, left shank)	88
I-P-RS	pressure insole; accelerometer (pelvis, right shank)	88
I-LS-RS	pressure insole; accelerometer (left shank, right shank)	88
I-H-P-LS	pressure insole; accelerometer (head, pelvis, left shank)	117
I-H-P-RS	pressure insole; accelerometer (head, pelvis, right shank)	117
I-H-LS-RS	pressure insole; accelerometer (head, left shank, right shank)	117
I-P-LS-RS	pressure insole; accelerometer (pelvis, left shank, right shank)	117
I-H-P-LS-RS	pressure insole; accelerometer (head, pelvis, left shank, right shank)	146

I: Pressure-sensing insole measures, H: Head accelerometer measures, P: Pelvis accelerometer measures, LS: Left shank accelerometer measures, RS: Right shank accelerometer measures.

**Fig 4:**
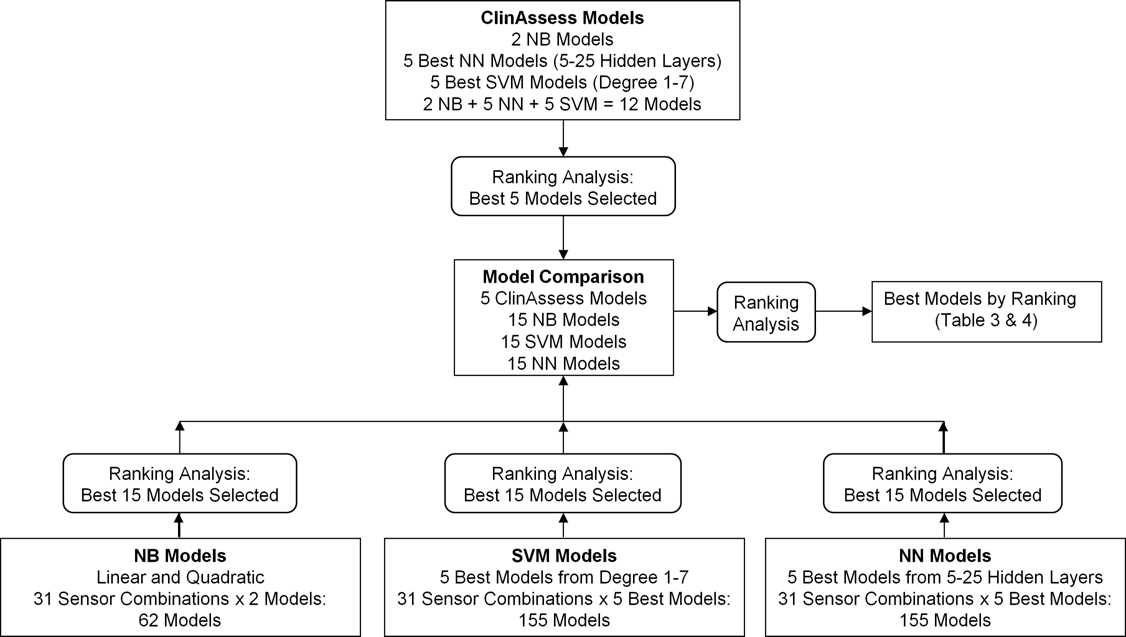
Model development and ranking analysis. ClinAssess: Clinical assessment measures, NB: Naive Bayesian, NN: Neural network, SVM: Support vector machine.

Model evaluation parameters included accuracy, specificity, sensitivity, positive predictive value (PPV), negative predictive value [40], F1 score (harmonic mean of precision and sensitivity) [41], and Matthew’s Correlation Coefficient (MCC) [42]. F1 score was calculated as:

$$F1=\frac{2PPV\cdot sensitivity}{PPV+sensitivity}=\frac{2TP}{2TP+FP+FN},$$ (1)

and MCC was calculated as:

$$MCC=\frac{TP\cdot TN-FP\cdot FN}{\sqrt{\left(TP+FP\right)\left(TP+FN\right)\left(TN+FP\right)\left(TN+FN\right)}},$$ (2)

where TP = true positive, TN = true negative, FP = false positive, and FN = false negative. A ranking method similar to Kendell [43] was used to determine the best models. Each model evaluation parameter was ranked from best (1) to worst (n), and ranks for all model evaluation parameters were summed to identify the overall best model (lowest summed rank) (Fig 4). For comparative purposes, classifying all participants as non-fallers would produce an accuracy of 76%, sensitivity of 0%, specificity of 100%, PPV of 0%, NPV of 76%, F1 score of 0, and MCC of 0.

## Results

Of the best 50 fall-risk classifier models based on ST data (Table 3), the top four models (I-P SVM, I-H-P SVM, I-P NN, I-H-P-LS NN) had identical top ranking scores with an accuracy of 84%, F1 0.600, and MCC 0.521. These models classified participants using support vector machines (degree 2 and 3) and neural networks (9 and 20 nodes) and included combinations of 30 pressure insole variables, 29 head accelerometer variables, 29 pelvis accelerometer variables, and 29 left shank accelerometer variables. The fifth best model (H SVM), based on 29 head accelerometer variables, achieved an overall accuracy of 84% and the highest scores for F1 (0.667) and MCC (0.561) but relatively low specificity and PPV prevented this model from ranking higher. The head sensor-based models ranked the highest of the single-sensor models with two models ranking in the top six. No other single-sensor models ranked among the top 10. All 50 models achieved an MCC > 0, indicating that their performance was better than chance. The five models based solely on clinical assessment data ranked the lowest.

**Table 3:** Best 50 fall risk classifier models based on ST gait data.

Sensors	Model Type	Accuracy (%)	Sensitivity (%)	Specificity (%)	PPV (%)	NPV (%)	F1	MCC	SR
I-P	SVM-2	84.0	50.0	94.7	75.0	85.7	0.600	0.521	49
I-H-P	SVM-3	84.0	50.0	94.7	75.0	85.7	0.600	0.521	49
I-P	NN-9	84.0	50.0	94.7	75.0	85.7	0.600	0.521	49
I-H-P-LS	NN-20	84.0	50.0	94.7	75.0	85.7	0.600	0.521	49
H	SVM-2	84.0	66.7	89.5	66.7	89.5	0.667	0.561	52
H	SVM-4	84.0	33.3	100.0	100.0	82.6	0.500	0.525	68
I-H	SVM-4	84.0	33.3	100.0	100.0	82.6	0.500	0.525	68
I-P-LS	SVM-2	84.0	33.3	100.0	100.0	82.6	0.500	0.525	68
H-P-LS-RS	NN-5	84.0	33.3	100.0	100.0	82.6	0.500	0.525	68
I-P-LS-RS	NB-Q	80.0	83.3	78.9	55.6	93.8	0.667	0.554	85
H	NB-Q	80.0	50.0	89.5	60.0	85.0	0.545	0.421	105
LS-RS	NN-23	80.0	50.0	89.5	60.0	85.0	0.545	0.421	105
I-P	NN-8	80.0	50.0	89.5	60.0	85.0	0.545	0.421	105
I-H-P-LS	NN-25	80.0	50.0	89.5	60.0	85.0	0.545	0.421	105
I-P	NB-Q	76.0	83.3	73.7	50.0	93.3	0.625	0.497	125
I-P-LS	NB-Q	76.0	83.3	73.7	50.0	93.3	0.625	0.497	125
H	SVM-6	80.0	33.3	94.7	66.7	81.8	0.444	0.369	132
H-P	SVM-3	80.0	33.3	94.7	66.7	81.8	0.444	0.369	132
I-H	SVM-2	80.0	33.3	94.7	66.7	81.8	0.444	0.369	132
I-H-P-LS	SVM-2	80.0	33.3	94.7	66.7	81.8	0.444	0.369	132
P	NN-5	80.0	33.3	94.7	66.7	81.8	0.444	0.369	132
P	NN-25	80.0	33.3	94.7	66.7	81.8	0.444	0.369	132
H-P	NN-20	80.0	33.3	94.7	66.7	81.8	0.444	0.369	132
LS-RS	NN-25	80.0	33.3	94.7	66.7	81.8	0.444	0.369	132
H-LS-RS	NN-15	80.0	33.3	94.7	66.7	81.8	0.444	0.369	132
P-LS-RS	NN-12	80.0	33.3	94.7	66.7	81.8	0.444	0.369	132
I-P-LS-RS	NN-21	80.0	33.3	94.7	66.7	81.8	0.444	0.369	132
I-P-RS	NB-Q	72.0	83.3	68.4	45.5	92.9	0.588	0.445	155
H-LS	NB-Q	76.0	50.0	84.2	50.0	84.2	0.500	0.342	170
H-P-LS	NB-Q	76.0	50.0	84.2	50.0	84.2	0.500	0.342	170
H-P-LS-RS	NB-Q	76.0	50.0	84.2	50.0	84.2	0.500	0.342	170
H	SVM-7	80.0	16.7	100.0	100.0	79.2	0.286	0.363	173
P	SVM-7	80.0	16.7	100.0	100.0	79.2	0.286	0.363	173
LS	SVM-1	80.0	16.7	100.0	100.0	79.2	0.286	0.363	173
H-P	SVM-5	80.0	16.7	100.0	100.0	79.2	0.286	0.363	173
H-LS	SVM-3	80.0	16.7	100.0	100.0	79.2	0.286	0.363	173
I-H-RS	NB-Q	68.0	66.7	68.4	40.0	86.7	0.500	0.306	205
I-P-LS	NB-L	68.0	66.7	68.4	40.0	86.7	0.500	0.306	205
H-P-RS	NB-Q	72.0	50.0	78.9	42.9	83.3	0.462	0.275	210
H-LS-RS	NB-Q	72.0	50.0	78.9	42.9	83.3	0.462	0.275	210
I-H-LS	NB-Q	72.0	50.0	78.9	42.9	83.3	0.462	0.275	210
I-H-P-LS	NB-Q	72.0	50.0	78.9	42.9	83.3	0.462	0.275	210
I-H-LS-RS	NB-Q	72.0	50.0	78.9	42.9	83.3	0.462	0.275	210
H	NN-15	76.0	33.3	89.5	50.0	81.0	0.400	0.266	233
P	NN-6	76.0	33.3	89.5	50.0	81.0	0.400	0.266	233
CA	NN-11	76.0	33.3	89.5	50.0	81.0	0.400	0.266	233
CA	NN-12	76.0	33.3	89.5	50.0	81.0	0.400	0.266	233
CA	NN-10	72.0	33.3	84.2	40.0	80.0	0.364	0.187	280
CA	SVM-1	72.0	16.7	89.5	33.3	77.3	0.222	0.081	305
CA	NN-9	72.0	16.7	89.5	33.3	77.3	0.222	0.081	305

SR: Summed Ranking, CA: Clinical assessment measures, I: Pressure-sensing insole measures, H: Head accelerometer measures, P: Pelvis accelerometer measures, LS: Left shank accelerometer measures, RS: Right shank accelerometer measures, NN: Neural network, NB: Naive Bayesian model, SVM: support vector machine, L: Linear, Q: Quadratic.

Of the best 50 fall-risk classifier models based on DT data (Table 4), the top model (I-P SVM) achieved an overall accuracy of 80%, F1 score of 0.706, and MCC of 0.634. This model classified participants using a support vector machine (degree of 1) and included 30 pressure insole variables and 29 pelvis accelerometer variables. The second best model, which was based solely on pelvis accelerometer data, achieved an overall accuracy of 80%, the second highest F1 score (0.545) and second highest MCC (0.421). This model classified participants using a neural network (7 nodes) and 29 pelvis accelerometer variables. All 50 models achieved an MCC > 0, indicating that their performance was better than chance. The pelvis sensor-based models ranked the highest of the single-sensor models, with three models ranking in the top ten. The next best single-sensor model was LS NN, with one model among the top 10. In contrast to the ST data, two of the models based solely on clinical assessment data ranked twelfth (identical scores).

**Table 4:** Best 50 fall-risk classifier models based on DT gait data.

Sensors	Model Type	Accuracy (%)	Sensitivity (%)	Specificity (%)	PPV (%)	NPV (%)	F1	MCC	SR
I-P	SVM-1	80.0	100.0	73.7	54.5	100.0	0.706	0.634	44
P	NN-7	80.0	50.0	89.5	60.0	85.0	0.545	0.421	45
P	NN-6	80.0	33.3	94.7	66.7	81.8	0.444	0.369	68
LS	NN-25	80.0	33.3	94.7	66.7	81.8	0.444	0.369	68
I-P	NN-14	80.0	33.3	94.7	66.7	81.8	0.444	0.369	68
I-P	NN-15	80.0	33.3	94.7	66.7	81.8	0.444	0.369	68
I-H-P	SVM-1	72.0	66.7	73.7	44.4	87.5	0.533	0.359	85
I-P-RS	SVM-1	72.0	66.7	73.7	44.4	87.5	0.533	0.359	85
I-P-LS	SVM-1	72.0	50.0	78.9	42.9	83.3	0.462	0.275	106
P	NN-10	72.0	50.0	78.9	42.9	83.3	0.462	0.275	106
I-P	NN-25	72.0	50.0	78.9	42.9	83.3	0.462	0.275	106
CA	NN-11	76.0	33.3	89.5	50.0	81.0	0.400	0.266	126
CA	NN-12	76.0	33.3	89.5	50.0	81.0	0.400	0.266	126
I-P	SVM-5	76.0	33.3	89.5	50.0	81.0	0.400	0.266	126
I-P	NN-13	76.0	33.3	89.5	50.0	81.0	0.400	0.266	126
I-LS	NN-9	76.0	33.3	89.5	50.0	81.0	0.400	0.266	126
I-H-P	NN-15	76.0	33.3	89.5	50.0	81.0	0.400	0.266	126
LS-RS	SVM-6	80.0	16.7	100.0	100.0	79.2	0.286	0.363	133
I-H-P-LS	NN-23	80.0	16.7	100.0	100.0	79.2	0.286	0.363	133
P	NB-L	60.0	66.7	57.9	33.3	84.6	0.444	0.210	143
H-P	NB-L	60.0	66.7	57.9	33.3	84.6	0.444	0.210	143
P	SVM-3	68.0	50.0	73.7	37.5	82.4	0.429	0.217	164
LS	SVM-3	68.0	50.0	73.7	37.5	82.4	0.429	0.217	164
P-RS	SVM-1	68.0	50.0	73.7	37.5	82.4	0.429	0.217	164
P-LS	NB-L	56.0	66.7	52.6	30.8	83.3	0.421	0.165	176
P-RS	NB-L	56.0	66.7	52.6	30.8	83.3	0.421	0.165	176
H-P-LS	NB-L	56.0	66.7	52.6	30.8	83.3	0.421	0.165	176
H-P-RS	NB-L	56.0	66.7	52.6	30.8	83.3	0.421	0.165	176
P-LS-RS	NB-L	56.0	66.7	52.6	30.8	83.3	0.421	0.165	176
H-P-LS-RS	NB-L	56.0	66.7	52.6	30.8	83.3	0.421	0.165	176
I-P	NB-L	56.0	66.7	52.6	30.8	83.3	0.421	0.165	176
I-H-P	NB-L	56.0	66.7	52.6	30.8	83.3	0.421	0.165	176
I-P-LS	NB-L	56.0	66.7	52.6	30.8	83.3	0.421	0.165	176
I-P-RS	NB-L	56.0	66.7	52.6	30.8	83.3	0.421	0.165	176
I-H-P-LS	NB-L	56.0	66.7	52.6	30.8	83.3	0.421	0.165	176
I-P-LS-RS	NB-L	56.0	66.7	52.6	30.8	83.3	0.421	0.165	176
P-LS	NN-5	76.0	16.7	94.7	50.0	78.3	0.250	0.180	180
I-H	NN-7	76.0	16.7	94.7	50.0	78.3	0.250	0.180	180
I-LS	NN-5	76.0	16.7	94.7	50.0	78.3	0.250	0.180	180
I-H-LS	NN-9	76.0	16.7	94.7	50.0	78.3	0.250	0.180	180
CA	NN-10	72.0	33.3	84.2	40.0	80.0	0.364	0.187	184
P	SVM-1	72.0	33.3	84.2	40.0	80.0	0.364	0.187	184
I-P	SVM-3	72.0	33.3	84.2	40.0	80.0	0.364	0.187	184
I-P-LS	SVM-3	72.0	33.3	84.2	40.0	80.0	0.364	0.187	184
P	SVM-5	68.0	33.3	78.9	33.3	78.9	0.333	0.123	237
RS	SVM-1	68.0	33.3	78.9	33.3	78.9	0.333	0.123	237
RS	SVM-2	68.0	33.3	78.9	33.3	78.9	0.333	0.123	237
CA	SVM-1	72.0	16.7	89.5	33.3	77.3	0.222	0.081	247
CA	NN-9	72.0	16.7	89.5	33.3	77.3	0.222	0.081	247
I	NB-Q	72.0	16.7	89.5	33.3	77.3	0.222	0.081	247

SR: Summed Ranking, CA: Clinical assessment measures, I: Pressure-sensing insole measures, H: Head accelerometer measures, P: Pelvis accelerometer measures, LS: Left shank accelerometer measures, RS: Right shank accelerometer measures, NN: Neural network, NB: Naive Bayesian model, SVM: support vector machine, L: Linear, Q: Quadratic.

A comparison between the ten best ST and ten best DT models (Table 5) shows that all but one of the ST models outranked and thus clearly outperformed the DT models.

**Table 5:** Comparison across 10 best ST and 10 best DT gait based models.

Gait Data	Sensors	Model Type	Accuracy (%)	Sensitivity (%)	Specificity (%)	PPV (%)	NPV (%)	F1	MCC	SR
ST	H	SVM-2	84.0	66.7	89.5	66.7	89.5	0.667	0.561	33
ST	I-P	SVM-2	84.0	50.0	94.7	75.0	85.7	0.600	0.521	35
ST	I-H-P	SVM-3	84.0	50.0	94.7	75.0	85.7	0.600	0.521	35
ST	I-P	NN-9	84.0	50.0	94.7	75.0	85.7	0.600	0.521	35
ST	I-H-P-LS	NN-20	84.0	50.0	94.7	75.0	85.7	0.600	0.521	35
ST	H	SVM-4	84.0	33.3	100.0	100.0	82.6	0.500	0.525	44
ST	I-H	SVM-4	84.0	33.3	100.0	100.0	82.6	0.500	0.525	44
ST	I-P-LS	SVM-2	84.0	33.3	100.0	100.0	82.6	0.500	0.525	44
ST	H-P-LS-RS	NN-5	84.0	33.3	100.0	100.0	82.6	0.500	0.525	44
DT	I-P	SVM-1	80.0	100.0	73.7	54.5	100.0	0.706	0.634	48
ST	I-P-LS-RS	NB-Q	80.0	83.3	78.9	55.6	93.8	0.667	0.554	49
DT	P	NN-7	80.0	50.0	89.5	60.0	85.0	0.545	0.421	73
DT	P	NN-6	80.0	33.3	94.7	66.7	81.8	0.444	0.369	84
DT	LS	NN-25	80.0	33.3	94.7	66.7	81.8	0.444	0.369	84
DT	I-P	NN-14	80.0	33.3	94.7	66.7	81.8	0.444	0.369	84
DT	I-P	NN-15	80.0	33.3	94.7	66.7	81.8	0.444	0.369	84
DT	I-H-P	SVM-1	72.0	66.7	73.7	44.4	87.5	0.533	0.359	85
DT	I-P-RS	SVM-1	72.0	66.7	73.7	44.4	87.5	0.533	0.359	85
DT	I-P-LS	SVM-1	72.0	50.0	78.9	42.9	83.3	0.462	0.275	102
DT	P	NN-10	72.0	50.0	78.9	42.9	83.3	0.462	0.275	102

SR: Summed Ranking, CA: Clinical assessment measures, I: Pressure-sensing insole measures, H: Head accelerometer measures, P: Pelvis accelerometer measures, LS: Left shank accelerometer measures, RS: Right shank accelerometer measures, NN: Neural network, NB: Naive Bayesian model, SVM: support vector machine, L: Linear, Q:Quadratic, ST: Single-task gait, DT: Dual-task gait.

## Discussion

Models derived from this investigation predicted retrospective fall occurrence with varying degrees of accuracy, sensitivity, and specificity. The large number of models assessed using different combinations of sensor-based-measures, model types, and ST or DT gait data permitted determination of the optimal combination for fall risk classification.

The head and pelvis accelerometers provided the best single-sensor classification capability, with two head sensor-based models ranking among the top six for ST and three pelvis sensor-based models among the top ten for DT. In previous studies, the pelvis or lower back location was the most frequent sensor site for fall risk prediction and classification models [6]. This location is intuitively appropriate since it is close to the body center of mass. The pelvis location also allows unobtrusive and easy monitoring with a belt attached sensor or accelerometer-equipped smartphone, and high user acceptance was found for a 20 day case-study with a lower back sensor [44]. The head location may have provided strong single-sensor results because it provided measurements relevant to visual input and upper body stability. While the head accelerometer performed well for ST gait assessment, the pelvis accelerometer performed better for DT gait assessment. The pelvis accelerometer appears in nine of the top ten DT models and seven of the top ten ST models, whereas the head accelerometer appears in one of the top ten DT models and six of the top ten ST models. The head accelerometer may not perform as well under DT conditions when the head may experience non-gait related movements during attention demanding periods (e.g., struggling to think of another word that starts with the desired letter, researcher prompts to continue with cognitive task). The pelvis location is less likely to experience non-gait related movements under DT conditions. Our study is the first to directly compare sensor locations (head, pelvis, left shank, right shank) to show that an accelerometer located at the head and posterior pelvis are superior for single-sensor-based fall risk classification.

While a single sensor is practical, the best results were found with multiple sensors, particularly when combining pelvis and head accelerometer with pressure-sensing insole parameters. The top ST models (I-P, I-H-P, I-P, I-H-P-LS) achieved an accuracy of 84%, F1 score of 0.600, MCC of 0.521, sensitivity of 50%, and specificity of 95% using the pressure-sensing insole and head, pelvis, and left shank accelerometers. The best single-sensor head-based model also achieved an accuracy of 84%, F1 score of 0.667, MCC of 0.561, sensitivity of 67%, and specificity of 90%. Therefore, the multi-sensor models were better at classifying non-fallers and the head-based model was better at classifying fallers. While the multi-sensor models ranked first in the ST ranking analysis (Table 3), the head-based model ranked first when comparing ST and DT models (Table 5), with all these models having similar SR scores. Therefore, both the multi-sensor and single-sensor models achieved strong fall risk classification performance and represent a trade-off between model sensitivity and specificity. The benefit of using insole and accelerometer sensor types, as well as multiple sensor locations, may outweigh the additional cost and complexity in implementing multiple sensors for point-of-care assessments. However, the head sensor accurately identified retrospective fall occurrence and should be considered if a lower cost and faster to implement assessment is desired.

ST models outperformed DT models in overall ranking of performance measures, thus demonstrating better fall risk classification by ST models. While DT gait can reveal increased fall risk from impaired executive functioning that impacts mobility control [45–48], fall risk has a broad spectrum of physical, psychological, social, and environmental risk factors [49]. DT gait data could improve classification ability for those with impaired executive functioning, but worsen classification ability for those with normal executive functioning. Furthermore, other studies have failed to find an improvement in fall prediction and classification under DT gait conditions, compared to ST, in older individuals [50,51]. For people at risk of falling but with normal executive functioning, normal gait deterioration due to a second cognitive task may mask gait-related fall risk factors, thus worsening fall risk classification performance.

The sensor based models were also compared to models developed from commonly performed clinical point-of-care assessments. ST sensor based models outperformed clinical assessment based models, with clinical assessment models ranking lowest of the 50 ranked models. These results demonstrate the advantage of using wearable sensors when assessing fall risk compared to using only common clinical assessments. This is supported by Weiss [16], van Schooten [17], and Rispens [18] who found that sensor-based classifier and predictive models, or a combination of sensor and clinical assessment, improved fall risk classification and prediction compared to clinical assessment alone.

Three different intelligent modeling techniques were assessed in this study: neural networks, naive Bayesian classifiers, and support vector machines. The top ten models, based on ST gait data, used six support vector machines, three multi-layer perceptron neural networks, and one naive Bayesian classifier (10th). Support vector machines and NN provided the best classification of retrospective fall occurrence when trained with ST gait-based data.

This study used retrospective fall occurrence as the criterion for classifying faller and non-faller status. While this is superior to using a clinical assessment based criterion [6], future studies should use prospective fall occurrence as the criterion for classification. Retrospective fall occurrence has two main limitations: inaccurate recall of falls and changes to gait patterns that occur between the fall and assessment, either in an attempt to increase stability or as a result of fear of falling. Future studies should use prospective fall occurrence. Future studies could include readily available participant information, such as age and sex, to determine if adding these parameters to the wearable sensor-based models improves performance.

In this study, computation time was considered acceptable since the run-time for trained models were typically less than 0.04 s for all models. The small computation time could provide outcome results to a clinician immediately after data collection. For applications that require computation times in the order of several ms, neural networks should be considered with caution since neural networks typically have longer computational time compared to support vector machines and naive Bayesian classifiers, given the greater complexity of neural networks [52].

## Conclusions

Wearable-sensor based models were able to predict retrospective fall occurrence in older individuals and outperform the predictive ability of models based on clinical assessments. Multi-sensor gait assessment provided the best input data for fall risk classification, using the foot-pressure-sensing insole and head, pelvis, and left shank accelerometers. Fall risk single-task gait assessment using a single-sensor would be best with the head sensor. Single-task gait assessment was better that dual-task for evaluating multi-factorial fall risk of older adults. Support vector machines and neural networks were the best intelligent modelling technique for fall risk classification. Fall risk classification models developed for point-of-care environments should be developed using support vector machines and neural networks, with a multi-sensor single-task gait assessment.
